# Intensity of Walking Training Impacts Cognition Among Assisted Living Residents With Frailty

**DOI:** 10.1093/geroni/igab046.1854

**Published:** 2021-12-17

**Authors:** Margaret Danilovich, Christie Norrick

**Affiliations:** 1 CJE SeniorLife, Skokie, Illinois, United States; 2 CJE SeniorLife, Chicago, Illinois, United States

## Abstract

Exercise has many proven cognitive benefits but the precise intensity to modify cognition is unclear. This pilot study investigated the role of exercise intensity on cognitive outcomes among assisted living residents. We enrolled n=33 frail or pre-frail residents who completed an 8 week, twice-weekly walking intervention. Participants were 66% female, and on average were 88 years old with a MMSE score=25.6, and low cognitive scores (Category Fluency Animals=10.45, Category Fluency Vegetables=7.67, Trail Making Test A=60.82 seconds, Trail Making Test B=155.18 seconds). Walking sessions used 5-minute intervals focused on maximizing steps, fast speeds, and multi-directions for 45 minutes per session. Participants in the high intensity group walked at >70% heart rate maximum or RPE 15-17 and those randomized to the casual intensity group walked at <60% heart rate maximum or RPE <13. Results showed the casual-intensity group improved more on Category Fluency tests (increase of 0.88 animals and 1.06 vegetables) compared to the high-intensity group (increase of 0.12 animals and increase of 0.35 vegetables). On Trail Making Test A, high-intensity participants had a 7.47 second decrease in time to complete compared to the casual-intensity group (2.00 seconds increase). On Trail Making Test B, high-intensity participants decreased time to complete by 27.13 seconds compared to a 26.19 decrease in the casual-intensity group. Results show promising trends in the role of exercise intensity in impacting different elements of cognition among assisted living residents.

